# Attention Deficits in Stroke Patients: The Role of Lesion Characteristics, Time from Stroke, and Concomitant Neuropsychological Deficits

**DOI:** 10.1155/2019/7835710

**Published:** 2019-05-23

**Authors:** Simona Spaccavento, Chiara Valeria Marinelli, Roberto Nardulli, Luigi Macchitella, Umberto Bivona, Laura Piccardi, Pierluigi Zoccolotti, Paola Angelelli

**Affiliations:** ^1^Neurorehabilitation Unit, ICS MAUGERI SPA SB, Institute of Cassano Murge, IRCCS, Cassano Murge, Italy; ^2^Department of History, Society and Human Studies, Lab of Applied Psychology and Intervention, University of Salento, Lecce, Italy; ^3^IRCCS Santa Lucia Foundation, Rome, Italy; ^4^Life, Health and Environmental Science Department, L'Aquila University, L'Aquila, Italy; ^5^Department of Psychology, University of Rome “Sapienza”, Italy

## Abstract

Attention impairments are frequent in stroke patients with important consequences on the rehabilitation outcomes and quality of life. The aim of the study was to perform a comprehensive assessment of selective and intensive attention processes in a large population of brain-damaged patients, evaluating the influence of the side and site of the brain lesion, the time from stroke, and the concomitant presence of aphasia or neglect. We assessed 204 patients with a first unilateral brain lesion and 42 healthy individuals with three subtests of the Test of Attentional Performance (TAP): Alertness, Go-No Go, and Divided Attention. 44.4% of patients had an impairment in both intensive and selective aspects of attention, 5.6% had deficits only in the intensive component, and 31.8% had deficits only in selective tasks. More than 80% of the patients fell below the cut-off point on at least one task. Patients with a right hemispheric lesion (RHL) were more impaired than patients with a left hemispheric lesion (LHL) especially in tonic and phasic alertness. Patients with total anterior infarcts (TACI) presented the worst profile compared to other stroke subtypes, with a difference between total and lacunar subtypes in the Alertness test, independent of the presence of warning. Patients in the chronic phase had shorter RTs than acute patients only in the Alertness test. In patients with LHL, the presence of aphasia was associated with a greater deficit in selective attention. In patients with RHL, the presence of unilateral neglect was associated with impaired alertness and selective attention. Attention deficits are common after a unilateral first stroke. In keeping with the hierarchical organization of attention functions, results confirm the important role of the right hemisphere for the intensive components of attention, also highlighting the involvement of left hemisphere functioning for the selective aspects, possibly indicating a role of its linguistic functions.

## 1. Introduction

Attention can be divided into two broad subsystems [1]: intensive processes, such as alertness and vigilance, and selective attention processes, such as focused and divided attention. The intensity aspects are probably a prerequisite for the more complex ones, such as selectivity [2].

Alertness refers to the condition of general wakefulness that enables a person to respond quickly and appropriately to any sudden given request for action. It is a prerequisite for effective behavior and, in this respect, is the basis of every attention performance. Alertness can phasically increase efficiency for a short period by both bottom-up (i.e., external stimulus) and top-down processes (i.e., internal factors; e.g., see [2–4]). Intrinsic (also called tonic) alertness refers to the cognitive (top-down) control of arousal and is typically assessed by simple reaction times (RT) to visual or auditory stimuli without a warning signal [3, 5]. Phasic alertness is the ability to increase the general level of attention for a short period in response to a cue, a warning stimulus in the same or a different sensory modality which precedes the target stimulus. Sustained attention has been defined as the ability to maintain a certain level of arousal and alertness over time; this is often referred to as “vigilance” in the case of protracted presentation of rare stimuli.

As it regards the selectivity aspects of attention processes, the model of van Zomeren and Brouwer [1] is distinguished between focused or selective attention and divided attention. Selective attention is the ability to focus attention on particular features of a task and to suppress reactions to irrelevant ones. The ability to perform two tasks simultaneously in the same, or in a different sensory modality, is defined as divided attention.

Attention deficits are quite common stroke-related deficits, and their occurrence is documented in several clinical studies. Attention deficits in stroke survivors are reported with a variable incidence ranging from 46% to 92% in different studies [6, 7]. The impairments described may regard different aspects of the attention process. In a sample of 94 stroke patients, Barker-Collo et al. [8] found an impairment only on a task evaluating selective and sustained attention, partially confirming the results of Hyndman and Ashburn [9] who showed a high incidence of divided (41%), selective (35%), and sustained (31%) attention deficits. In the study of McDowd et al. [10], divided and switching attentions were more impaired in stroke patients compared with those in control subjects.

The presence of attention disorders in stroke patients has important implications for the everyday functioning of these patients. In particular, these disorders constitute a serious obstacle to rehabilitation [7, 9]. They lead to greater functional impairment with a negative impact of attention deficits on daily functioning. Attention deficits, in fact, are associated with difficulties in balance, daily living activities, and falls [10].

Overall, the assessment of attentional deficits and the understanding of their clinical correlates are important in stroke survivors. However, although there is converging evidence on the high prevalence of attention deficits, it is not easy to draw a definite profile of attention deficits in stroke patients. Differences related to sampling variables (e.g., time from stroke, side of lesion, and type of stroke) or tasks (tapping the intensive or selective aspects of attention) may explain in part this variability. Furthermore, association to critical neuropsychological symptoms may also modulate attention performance.

As numerous studies have shown, attention functions can be selectively impaired as a function of the side of the lesion [3, 11–15]. Lesional and neuroimaging studies indicated that intrinsic and sustained attention processes involve a right hemisphere cortical and subcortical network [3] whereas phasic alertness also involves left hemispheric structures [3, 5, 12, 16]. However, some studies comparing patients with either a right-hemispheric lesion (RHL) or a left-hemispheric lesion (LHL) failed to find significant group differences in simple RTs, in phasic alertness and in sustained attention [11, 17]. Left hemisphere mechanisms are known to be involved also in several tasks (e.g., Stroop and Go-No Go tasks) and more complex attention functions, including selective, executive, and temporal attention [11–13, 15, 18].

While there has been a sizeable amount of research on the laterality of attention, clinical studies examining the intrahemispheric correlates of different attention deficits are much fewer. A useful classification to this aim, the Oxford Community Stroke Project classification (OCSP), was proposed by Bamford et al. [19]. The OCSP is a simple but reliable method of categorizing subacute ischemic stroke patients based on a large population study of first ever stroke [19]. This classification system is based on initial clinical symptoms and includes total anterior circulation infarcts (TACI), lacunar infarcts (LACI), partial anterior circulation infarcts (PACI), or posterior circulation infarcts (POCI). Many studies [e.g., [20, 21]] examined physical outcomes in relation to stroke subtype. TACI are associated with the greatest case fatality and poor functional outcomes [19, 22, 23]. However, to the best of our knowledge, attention deficits after a brain lesion have not yet been examined as a function the OCSP.

Moreover, the time from onset is an important variable that influences attention deficits. Patients with a longer time between stroke onset and assessment may have a better performance in some attention tasks [8] although there is also evidence that these deficits can persist over long periods of time after stroke [7, 9].

There is some evidence that attention deficits covary with other neuropsychological deficits, particularly aphasia and neglect. As to language deficits, Murray [24, 25] found that patients with aphasia showed more impairment in attention functions than the control group, but with the variability in the presence, type, and severity of the attention deficits. Zimmerman and Leclercq [26] assessed attention functions in a large sample of patients with aphasia. Compared to healthy controls of their age, patients with aphasia did not have frequent problems of alertness and the frequency of patients with an increased rate of false alarms in the Go-No Go task was also comparable to that of healthy subjects. However, patients with aphasia were impaired in divided attentions and also had higher RTs in the Go-No Go task. Zimmermann and Leclercq [26] stressed that the RTs of aphasic patients indicated a profound impairment in alertness in several patients. Therefore, the authors concluded that, even if alertness is not much more frequently impaired in aphasic patients compared to controls, assessing alertness is recommended since in single cases of profound reductions of activation level may influence more complex attentional aspects.

Patients with neglect commonly have low general arousal [27] with deficits in phasic alertness [28] and sustained attention [29, 30] as well as a significant decrease in vigilance over time [31].

Overall, the results of these studies show a complex picture with several different factors contributing to the individual performance in attention tasks. The aim of the present study was to provide a comprehensive evaluation of these factors within the same sample of patients. Thus, the presence of attention deficits, both in selective and in intensive aspects, was examined in a large sample of first stroke patients, relative to age-matched healthy adults, evaluating the role of lesion characteristics (such as the side of the hemispheric lesion and site of lesion), time from stroke, and the copresence of critical neuropsychological deficits (i.e., aphasia and neglect).

## 2. Methods

### 2.1. Sample

This was a multicenter study, enrolling all stroke patients consecutively admitted to two neurorehabilitation Units in the center and south of Italy (IRCCS Santa Lucia Foundation, Rome, and ICS Maugeri spa SB IRCCS, Cassano Murge-Bari). The neurological and neuropsychological criteria for inclusion (or exclusion) in the study were as follows:

1. *Neurological criteria*: all patients were suffering from the consequences of a unilateral cerebral ischemic stroke, documented by computerized tomography or magnetic resonance imaging data. We excluded patients with bilateral lesions, with a previous stroke, noncerebral involvement, or patients who had undergone surgery (e.g., for aneurysm). Patients with other chronic disabling pathologies (polyneuropathy, cancer, and limb amputation) or other central nervous system diseases were also excluded. Finally, patients were excluded if they had prior psychiatric/substance abuse histories
2. *Neuropsychological criteria*: all patients presented adequate sufficient levels of awareness, linguistic ability, and abstract reasoning to carry on the experimental tasks (see the following paragraph). Patients with severe anosognosia (as assessed by a structured questionnaire), severe comprehension deficit [performance on Token test < 10 (corrected score)], or cognitive decline [performance on Raven's Progressive Matrices test < 1 (equivalent score)] were excluded.

A total of 204 stroke patients fulfilled these criteria over a two-year period and were enrolled in the study. All patients were tested between two and twelve months after the onset of stroke. Dividing the patient's groups according to the side of hemispheric lesion, 108 (39 F and 69 M) had a RHL and 96 (39 F and 57 M) had a LHL.

Patients were compared to a group of 42 healthy controls (20 M and 22 F). They were recruited out of hospital and, as a group, were matched with stroke patients for gender, age, and education (all Fs < 1). For this group, the exclusion criteria included prior neurological or psychiatric disorders and recent reduction of cognitive efficiency, carried out by a careful neuropsychiatric evaluation done by a neuropsychologist.  Table 1 reports the demographic and clinical variables of the patients' and controls' groups.

The study was conducted according to the principles of the Helsinki Declaration and was approved by the local Ethical Committees of the participating centers. Each participant signed a consent form.

### 2.2. Neurological, Functional, and Neuropsychological Assessment

The Oxford Community Stroke Project classification (OCSP) [19] was used to categorize patients' stroke. Based on neuroradiological and clinical evidence, strokes were classified as follows: total anterior circulation infarcts (TACI; 35 patients, 17.2%); partial anterior circulation infarcts (PACI; 117 patients, 57.3%); lacunar infarcts (LACI; 45 patients, 22.1%); and posterior circulation infarcts (POCI; 7 patients, 3.4%).  Table 1 reports the percentage of patients with RHL and LBL suffering from the different types of stroke. None of the comparisons was significant.

The Functional Independence Measure (FIM) [32] was used to measure functional impairment. Additionally, both patients with RHL and LHL performed some selected cognitive and functional tests, including the Token test (normative data according to Spinnler and Tognoni [33]) and Raven's Progressive Matrices (normative data according to Basso et al. [34]). Significant differences were found in the Token test (*F*_(1,201)_ = 51.35, *p* < .0001), with patients with LHL showing greater impairment in verbal comprehension (mean accuracy = 24.1, with respect to 31.3 of patients with RHL). In Raven's Progressive Matrices, patients with RHL showed lower scores than patients with LHL (mean accuracy = 23.5 and 25.8, respectively; *F*_(1,201)_ = 11.91, *p* < .001). As to the FIM total score, patients with RHL showed more functional impairment than patients with LHL (mean accuracy = 83.2 and 91.7, respectively; *F*_(1,201)_ = 6.40, *p* < .01).

Neglect and language disorders were detected by a specific neuropsychological examination.

In patients with LHL, language disorders were analyzed by two standardized batteries for the examination of language deficits (language examination-II: [35]; Italian Version of the Aachen Aphasie Test: [36]). Among patients with LHL, 77 (80.2%) suffered from aphasia. Classification into the different aphasic syndromes is out of the scope of the present work; however, patients mainly presented difficulties in productive language, with 41.5% showing Broca's aphasia, 36.6% amnestic aphasia, 19.5% Wernicke's aphasia, and 2.4% transcortical sensory aphasia.

In patients with RHL, hemispatial neglect was assessed by the standardized battery for the evaluation of hemineglect [37] which includes four tests: Letter Cancellation Test, Line Cancellation Test, Wundt–Jastrow Area Illusion Test, and Sentence Reading Test. Following the norms of the test, patients were diagnosed as having unilateral neglect if they performed below the cut-off in at least two out of four tests. In patients with LHL, the presence of neglect was assessed by the star cancellation test, one of the subtest of the BIT (Behavioral Inattention Test [38]). Due to differences in the two neuro-rehabilitation units the assessment of neglect in patients with LHL was done only in 64 out of 108 patients. As reported in Table 1, among patients with RHL, 42 (65.6%) suffered from neglect. None of patients with LHL screened displayed neglect.

## 3. Materials

To assess attention deficits, we used three subtests from the Test of Attentional Performance (TAP) [39]: Alertness test, Go-No Go test, and Divided Attention test.

### 3.1. Alertness

This subtest measures the RTs to simple visual target with or without a warning signal (tone). A cross appears in the middle of the computer screen, and the subject has to press a button as rapidly as possible. The order of block presentation is ABBA, in which A is the block without a tone and B is the block with a warning signal. A total of 80 trials were presented to each participant. The median RTs of the subtests with and without warning were considered dependent measures.

### 3.2. Go-No Go

**Figure 1:**
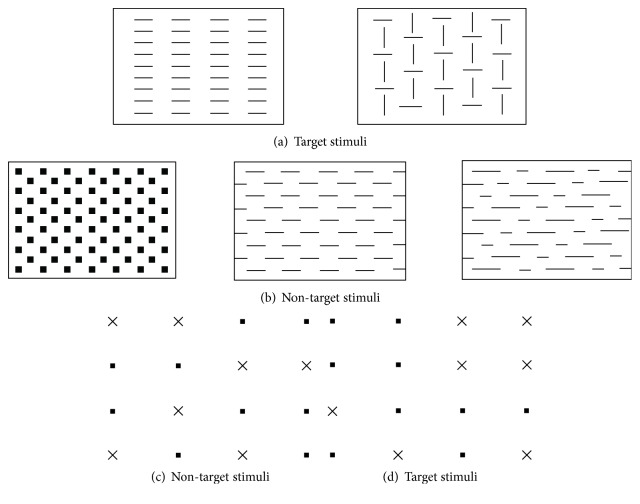
(a, b) Stimuli of the Go-No Go test. (c, d) Non-target and target stimuli in the Divided Attention test.

This subtest measures selective attention. One three by three cm square appears in the middle of the screen. There are two target and three nontarget stimuli (see Figures 1(a) and (b)). The subject has to press the button on the presentation of a target and not to press on the presentation of a nontarget. A total of 60 trials were presented. The main parameters were RTs for correct responses and number of false reactions.

### 3.3. Divided Attention

Two tasks, one visual and one auditory, are presented simultaneously. In the visual task, a matrix of sixteen dots (4 × 4) with seven little “x's” are displayed on the screen (see Figure 1(c)). The subjects have to press a key when four “x's” form a square (see an example in Figure 1(d)) and refrain from pressing they do not (see Figure 1(c)). In the auditory task, a series of two sounds, one high and one low, is presented (Di-Da-Di-Da, etc.): the task is to detect a variation in the sequence (Di-Di or Da-Da). RTs and the number of omissions were the measures considered.

## 4. Procedure

Participants were tested individually in a quiet room. Stimuli were presented on the screen of a PC computer about 60 cm away from the patient. Patients responded by pressing one button connected with the PC, thus allowing the measurement of RTs and errors (i.e., number of false responses or omissions). Instructions for each test were given aloud, and a brief sequence of practice trials preceded each test.

Note that, due to technical problems, accuracy data (false reactions in the Go-No Go test and omissions in the Divided Attention test) were missed for 37% of patients with LHL and 32% of patients with RHL.

## 5. Data Analysis

Following the indications of the TAP battery, the median RTs of the Alertness, Go-No Go, and Divided Attention tests were corrected for age and education. The general form of this formula is shown as follows:

(1) $$\begin{array}{c} \text{Corrected score}=\left\{\text{raw score}–\left[\left(\text{age}–41.34\right)*\left(\text{correction value}\right)\right]\right\}, \end{array}$$

Correction values vary for the different tests [39]. Limited to the Go-No Go sub-test, there is also an additional correction for years of schooling: 13 ms is added to the scores of patients with 12 years of schooling or more while 4 ms is subtracted for patients with less than 12 years of schooling. Corrected scores allow comparing patients' data to standardized norms considering a pathological performance below the 5th percentile on the normative sample [39]. No correction is envisaged for the error scores.

First, we examined the percentage of patients unable to perform the different attention tests. Then, we computed the proportion of patients with a pathological performance, i.e., performing at or below the fifth percentile according to Zimmermann and Fimm's [39] normative data. The frequency of pathological performance was compared for patients with RHL and LHL and for patients with concomitant neuropsychological deficits (i.e., aphasia or neglect) by chi-square tests.

Univariate analyses of variance were carried out to compare RTs and errors as a function of lesion laterality (i.e., left and right), lesion classification (TACI, LACI, and PACI), and time from stroke and presence of concomitant neuropsychological deficits (aphasia or neglect). To more directly test the effect of age and education on patient's performance, analyses were carried out on raw (uncorrected) data taking into consideration age and years of schooling as covariates. However, for the sake of presentation, the mean values reported in the text always refer to corrected scores. To evaluate the effect of distance from stroke, we divided the overall group in subacute (<90 days) and chronic (>90 days) patients. There were 123 patients in the subacute phase and 81 in the chronic phase.

## 6. Results

Table 2 reports the percentage of patients performing at or below the fifth percentile based on the normative data for the whole group of patients and for the patients with LHL and RHL.

In the whole sample, 44.4% of patients had impairment in both intensive and selective aspects of attention, with a pathological speed performance in all tasks; 5.6% had deficit in only intensive component (Alertness with and without warning) and 31.8% only in selective tasks (Go-No Go and Divided Attention). Thus, overall, 81.8% of patients fell in at least one attentional measure while 18.2% showed no attention deficits. Incidence of pathological speed performances was similar in intensive vs. selective attention tests: nearly half of the patients fell below the cut-off point on the Alertness test with and without warning (45% and 44%) and on the Go-No Go (47%) and Divided Attention (40%) tests. Regarding error data, 71% of patients displayed omissions in the Divided Attention test and 41% made false reactions in the Go-No Go test.

Comparisons between patients with LHL and RHL (Table 2) revealed a significantly higher incidence of pathological cases among the patients with RHL for the two measures of Alertness, with and without warning (*p* < .001 and *p* < .0001, respectively), and a comparable proportion of patients with impaired RTs in the Go-No Go and Divided Attention tests in the two groups. Regarding error data, the percentage of patients displaying a pathological rate of omissions in the Divided Attention test was higher in patients with RHL than in patients with LHL (*p* < .0001); the two groups did not differ in the proportion of patients with a pathological rate of false responses in the Go-No Go test.

Table 3 presents the means (and SDs) of RTs and accuracy data for patients with LHL and RHL and controls. In the Alertness test, the ANOVA revealed a main effect of group (*F*_(2,244)_ = 7.78, *p* < .001), indicating slower RTs in patients with RHL with respect to controls (*p* < .001) and patients with LHL (*p* < .05). Patients with LHL and controls did not differ from each other. The main effect of warning approached a significance (*F*_(2,244)_ = 2.68, *p* = .10), with shorter RTs in the warning condition (339 ms) with respect to the no-warning condition (353 ms). The group by warning interaction was not significant (*F* < 1), indicating no group difference in the warning effect in the two groups. As for covariates, only the age by warning interaction was significant (*F*_(1,244)_ = 7.52, *p* < .01).

In the Go-No Go test, the main effect of the group was significant for both RTs (*F*_(2,244)_ = 8.34, *p* < .001) and false responses (*F*_(2,179)_ = 4.81, *p* < .01). With regard to RTs, healthy controls were faster than both patients with RHL (*p* < .0001) and LHL (*p* < .0001), while the two groups of patients did not differ from each other. With regard to false alarms, patients with RHL differed from healthy subjects (*p* < .0001), while patients with LHL did not differ from patients with RHL or healthy controls. As for covariates, only years of schooling modulated the performance in the Go-No Go test: the effect was significant in the analysis on false alarms (*F*_(1,179)_ = 9.86, *p* < .01) and approached significance in the analysis on RT data (*F*_(1,236)_ = 3.66, *s*).

In the Divided Attention test, the effect of the group was significant for RTs (*F*_(2,224)_ = 12.04, *p* < .0001) and omissions (*F*_(2,179)_ = 27.96, *p* < .01). Healthy controls were faster than both patients with RHL (*p* < .0001) and LHL (*p* < .01); there was a tendency for patients with RHL to react more slowly than patients with LHL (*p* = .052). With regard to errors, patients with RHL committed more omissions than healthy controls (*p* < .0001) and patients with LHL (*p* < .0001), while patients with LHL made a comparable number of omissions than controls. The years of schooling covariate influenced the performance in the Divided Attention test: the effect was significant in the case of omissions (*F*_(1,179)_ = 27.96, *p* < .0001) and approached significance in the case of RT data (*F*_(1,236)_ = 3.05, *p* = .08).

Comments: As for Alertness, only patients with RHL (but not those with LHL) were slower than healthy controls independent of the presence of warning. This pattern held true both in terms of mean performance and incidence of individual patients performing below the cut-off. RTs in performing the Go-No Go and Divided Attention tests were impaired not only in patients with RHL but also in patients with LHL; again, this pattern emerged both at a group and at an individual level. In the Go-No Go test, the two groups did not differ. However, it should be noted that several LBD patients with LHL were unable to carry out this test while this occurred quite rarely among patients with RHL. In the Divided Attention test, incidence of pathological performance was similar but patients with RHL showed more omissions than patients with LHL and also tended to be slower. Note that a sizeable proportion of patients with both RHL and LHL was unable to perform this test.

Overall, both mean data and individual analyses highlighted a specific pattern of attention impairment, with patients with RHL suffering from more severe and frequent deficits in intensive attention as well as divided attention and patients with LHL presenting frequent failures in selective attention_._

### 6.1. Influence of OCSP Classification on Attention Measures

**Figure 2:**
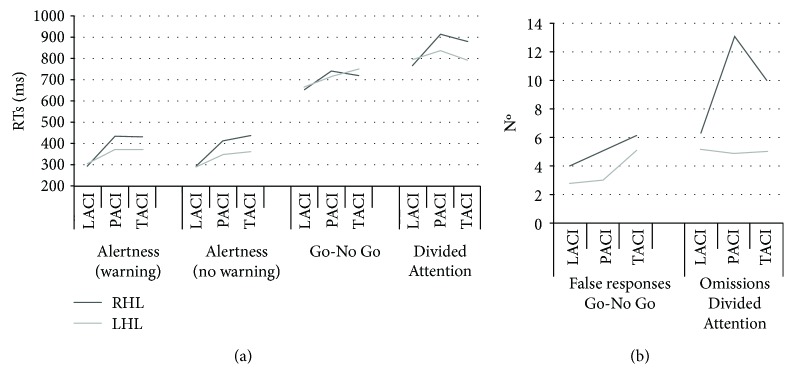
RTs (a) and errors (b) on each attention measure as a function of side and of the site (based on the OCSP classification) of the lesion.

Performance on each attention measure was examined as the function of laterality and OCSP classification. As shown in Figures 2(a) and 2(b) (RTs and error measures, respectively), patients with LACI showed generally better attention performance with respect to the other groups in all tests; patients with PACI and TACI were more impaired especially following RHL. Patients with POCI were very few in both the LHL and RHL groups (4 and 3 patients, respectively); for this reason, data for this subgroup were not considered in statistical analyses. At any rate, inspection of data indicated that patients with POCI showed a profile similar to that of patients with LACI in Alertness and Divided Attention tests with slower RTs in selective attention.

The role of lesional variables was analyzed with ANOVAs with lesion side (RHL and LHL) and lesion site (i.e., PACI, LACI, TACI) as unrepeated factors, separately for each attention measure.

In the case of Alertness, the lesion site effect was significant (*F*_(2,189)_ = 7.66, *p* < .001), indicating that patients with LACI had shorter RTs (300 ms) than those with PACI (394 ms, *p* < .001) and TACI (396 ms, *p* < .01) who did not differ from each other. The lesion side effect approached significance (*F*_(1,189)_ = 2.99, *p* = .08), indicating shorter RTs for patients with LHL (341 ms) than patients with RHL (386 ms). The warning effect approached significance (*F*_(2,189)_ = 3.13, *p* = .08), indicating shorter RTs in the warning with respect to the no-warning condition (358 ms vs. 369 ms, respectively). As for covariates, only the age by warning interaction was significant (*F*_(1,189)_ = 8.21, *p* < .01).

In the Go-No Go test, the analysis for RTs indicated the significance of the lesion site factor (*F*_(2,181)_ = 4.09, *p* < .05), with faster RTs in patients with LACI (660 ms) with respect to the other groups (about 730 ms, *p* < .05). None of the covariates are proven significant. In the analysis on false alarms, no main effect or interaction are proven significant, except for the year of schooling covariate (*F*_(1,126)_ = 7.66, *p* < .01).

In the Divided Attention test, the ANOVA on RTs indicated a significant main effect of the *lesion site* factor (*F*_(2,170)_ = 5.27, *p* < .01), indicating shorter latencies for patients with LACI (778 ms) with respect to patients with PACI (879 ms, *p* < .01) patients; patients with TACI had intermediate RTs (835 ms) not differing from either of the other two groups. None of the covariates are proven significant. The analysis on omissions indicated both a main effect of the lesion side (*F*_(1,121)_ = 10.14, *p* < .001) and *site* (*F*_(2,121)_ = 4.01, *p* < .05) factors. The *lesion side* by *lesion site* interaction approached significance (*F*_(2,121)_ = 2.35, *p* = .09). In patients with RHL, the number of omissions was higher for patients with PACI (13.11) than for patients with LACI (6.39, *p* < .001) but not for patients with TACI (10.00, n.s.); in patients with LHL, omissions were generally low and did not vary as a function of the lesion site (see Figure 2). Only the years of schooling covariate are proven significant (*F*_(1,179)_ = 5.44, *p* < .05).

Comments: patients with lacunar infarcts presented generally better performance in all tests while patients with partial or total anterior circulation infarcts showed more severe deficits. Patients with PACI and TACI were slower in each attention measure while not differing from each other. The same pattern of results (with better performance in patients with LACI with respect to those with PACI or TACI) was found in terms of omissions in the Divided Attention test but only in the group with RHL; for patients with LHL, no difference was found as a function of the lesion site. For false responses in the Go-No Go test, neither lesion site nor laterality affected the performance.

### 6.2. Differences between Chronic and Acute Patients

In the Alertness test, the stroke onset factor was not significant (*F*_(1,200)_ = 2.04, n.s.), as well as the warning factor (*F*_(1,200)_ = 2.53, n.s.): patients in the subacute phase employed on average 334 ms while patients in the chronic phase 296 ms. The age by warning covariate was significant (*F*_(1,200)_ = 6.78, *p* < .01).

In the Go-No Go task, the stroke onset factor was not significant for RTs (*F*_(1,195)_ = .27, n.s.), with very similar RTs in the two groups (641 and 643 ms for patients in the subacute and chronic phases, respectively) and for number of false response (*F*_(1,135)_ = 1.64, n.s.) (4.6% in the subacute group and 3.0% in the chronic one). As for covariates, years of schooling were significant only in false response data (*F*_(1,135)_ = 8.55, *p* < .01).

The stroke onset factor was not significant in the case of the Divided Attention test for both RTs (*F*_(1,183)_ = 2.76, n.s.) and omissions (*F*_(1,129)_ = 1.46, n.s.): in the subacute phase, patients had mean RTs of 695 ms with 8.0% of omissions while, in the chronic phase, patients had mean RTs of 644 ms with 8.7% of omissions. Covariates were significant only in the case of omission data (age: *F*_(1,129)_ = 5.79, *p* < .05; years of schooling: *F*_(1,129)_ = 7.69, *p* < .01).

### 6.3. Attention and Concomitant Cognitive Deficits

Table 4 presents the proportion of patients with and without aphasia (as well as neglect) showing a performance below the cut-off in the various tests while Table 5 reports the group data.

As it regards linguistic deficits, the presence of aphasia was associated to a higher incidence of patients underperforming in the Go-No Go test (Table 4); a trend was also present for aphasic patients to show more impaired performance in the Alertness with warning task.

As for group trends (Table 5), patients with aphasia tended to be slower than patients without aphasia in the Go-No Go test.

Relative to the presence of neglect, exploration of individual data (Table 4) shows that neglect was associated with a higher number of pathological cases both in intensive attention (Alertness with and without warning; *p* < .05 and *p* < .01, respectively) and for the Go-No Go test (*p* < .01). In terms of group data (Table 5), the presence of neglect was associated to slower RTs in the Alertness and the Go-No Go tests and to more omissions in the Divided Attention test.

## 7. Discussion

In the literature, attention deficits in stroke survivors are reported in a range from 46% to 92% in different studies [6–8]. Our results confirm the high incidence of attention deficits in a large sample of stroke patients: in fact, more than 80% of patients suffered from a significant impairment in at least one attention task. Note that, in order to evaluate the proportion of pathological patients, we adopted a test largely used in the neuropsychological clinic with solid normative data allowing correction for the influence of age and education. However, in order to examine the effect of age and education on the variable object of our study, we carried out analyses on raw data partialling out these variables as covariates. Results highlighted that age and education significantly modulated attentional performance, in particular with regard to the effect of lesion laterality (i.e., left and right), lesion classification (TACI, LACI, and PACI), and time from stroke, but not in the case of the effect of concomitant neuropsychological deficits (aphasia or neglect). In general, these data confirm the importance that in a clinical setting, raw data are corrected for age and education of the participant in order to control for the modulating role of these variables.

In keeping with the multicomponential organization of attention functions proposed by van Zomeren and Brouwer [1], some patients had deficits only in intensive components of attention while other patients experienced impairments in only selective aspects of attention. However, the largest proportion of patients (44.4%) actually showed a deficit in both intensive and selective aspects of attention, possibly indicating their partial interdependency.

As to the role of laterality of lesion, the results indicated that patients with RHL suffered from a greater impairment in attention functions: they showed slower RTs and more errors and had a higher incidence of pathological cases than patients with LHL in almost all tasks. Anatomical and functional studies are in keeping with these results. Evidence indicates that the right hemisphere plays a crucial role in maintaining and controlling intensity aspects of attention [3, 4, 40]. The specialization of the right hemisphere appears to guide the deployment of attention as well as the maintenance of sustained attention [41, 42]. In particular, studies on stroke patients revealed an important role of the right hemisphere in alertness [43, 44]. Moreover, lesion studies have showed that it was intrinsic alertness and not phasic alertness to be compromised after right hemisphere damage [16], and neuroimaging studies revealed that the left hemisphere plays a role in phasic alertness [3, 5, 12, 45]. In keeping with this idea, in our study, the deficit in alertness in patients with RHL did not interact with the presence/absence of a warning signal. This indicates that the sensitivity to the presence of a warning signal is preserved in these patients and that the deficit is largely due to the tonic component of alertness.

In focused and divided attention tasks, Nebel et al. [46] found an activation mainly of a right-sided network including dorso- and ventrolateral prefrontal structures, superior and inferior parietal cortex, and anterior cingulated gyrus. Under higher cognitive demands of divided attention, activity in these structures was enhanced and left-sided homologues were recruited. Accordingly, in the present study, comparisons between patients with RHL and LHL revealed no difference in the incidence of patients underperforming on the Go-No Go and Divided Attention tests, even though the group analysis showed more severe impairment in the divided attention in patients with RHL with respect to those with LHL. Indeed, our data indicate that both hemispheres are involved in selective and divided attention. Thus, in keeping with previous findings [2, 13, 47, 48], our study pointed out the involvement of the left hemisphere in selective attention.

There are few studies in the literature that evaluated the characteristics of neuropsychological deficits according to the OCSP classification. Sturm et al. [49] assessed the handicap, i.e., the disadvantage for an individual resulting from an impairment or disability that limits the fulfillment of a role that is normal for the subject. They found that patients with TACI were more handicapped than those with other subtypes of stroke. On cognitive aspects, Barker-Collo et al. [50] showed that survivors with TACI had the greatest impairment, particularly in visual organization, problem solving tasks, and word finding, while PACI was associated with the best cognitive profile.

The present data on attention skills are in keeping with these tendencies, as patients with TACI were more impaired in all attention tasks compared to the other groups. Even though composed by few patients, the group with POCI was the fastest. These results are compatible with the important role of the frontal lobe (as well as with its cerebral extension) in attention functioning which was impaired in both patients with total and partial anterior strokes. These data reflect the literature relating OCSP stroke subtypes to outcomes, which states that TACI have the worst outcomes with regard to mortality, quality of life, participation, and activity limitations.

For what regards distance from stroke, data did not indicate sizeable differences between subacute and chronic patients for both intensive and selective attention functions. These findings are in line with the results of Hyndman [7] who reported that, while some reduction may be detected in the early stages after stroke, attention deficits tend to persist over time representing an important sequela of the cerebral lesion.

We also examined the possible association of attention deficits to key neuropsychological symptoms. Results showed that the presence of aphasia was associated with deficits in selective attention. In particular, patients with aphasia were more impaired in selective functions than patients without aphasia. A review of the literature showed a cooccurrence of attention deficits and aphasia [51–53], even though these studies investigated only one or a restricted range of attention functions. Recently, Villard Kiran [54] found a significant effect of task complexity on RTs in a group of patients with aphasia, compared with controls. Murray [25] found a correlation between auditory comprehension, spoken language and communication abilities, and attention measures in patients with aphasia. Complex attention abilities were more strongly associated with language and communications deficits. The attention allocation difficulties could negatively affect auditory comprehension, or the patient's aphasic symptoms could influence the performance on attention tasks. The above findings suggest that language could affect attention. The relation between aphasia and selective attention could be mediated by language-related working memory (WM) processes. One of the WM components, the subvocal rehearsal loop, provides assistance in sustaining verbal information in working memory to be used in subsequent actions. Many studies showed that in tasks of articulatory suppression, the subject's performance is badly affected with slower RTs and/or an increase in the number of erroneous responses [55, 56]. Verbal strategies are used to aid performance when a high level of competition between tasks is expected [55]. A number of recent studies indicate that working memory representations can serve selective attention (for a review, see [57]). Laures-Gore et al. [58], comparing the performance of individuals with LBD and aphasia with those of individuals with RBD on verbal working memory, found that the differences between RBD and LBD may be explained by decreased attentional capacity or inefficient resource allocation in patients with aphasia.

In our study, we paid attention to select attention tasks including only nonverbal stimuli and then should be not influenced by language disease of LBD patients. However, the patients with aphasia in the Go-No Go test were more impaired than patients without aphasia. Furthermore, many researchers showed that even the short-term retention of abstract shapes is sensitive to verbal distraction [59]. High levels of similarity between visual stimuli increased the need for internal verbalization to stabilize perceptive discrimination. According to the “verbal-loop hypothesis,” unfamiliar abstract visual material requires more complex verbal codes and aphasic patients present even greater difficulty with these. Our results could be explained by this hypothesis: the Go-No Go visual stimuli could demand a verbal encoding in order to discriminate them; this ability is impaired in patients with aphasia causing a slowing down of reaction times. Another hypothesis could be that language is implicated in self-control and inhibition processes involved in a selection process. Finally, the higher attentional impairment in aphasic patients among the group with LHL might depend on a larger lesion site among aphasic patients with respect to nonaphasic one.

The present study also examined attention skills in relation to neglect. Our data are in agreement with previous studies indicating a close association between nonspatial attention deficits and neglect following RHL [e.g., [42]]. In fact, an impairment of alertness is a common observation in patients with neglect [27], due also to an overlap between the cerebral networks underlying tonic and phasic alertness and those involved in governing spatial attention [4, 60]. Patients with neglect show low general arousal [27], marked deficits in sustaining attention [29, 30], a significant decrease in vigilance over time [31], and deficits in phasic alertness [28]. According to Kerkhoff [61], the selective specialization of the right hemisphere in arranging attentional resources may account for the clinical observation of more frequent, severe, and long-lasting neglect following RHL. As already found by Robertson et al. [30], patients with RHL suffering from neglect were significantly less accurate in tone counting than patients without neglect. These authors report a high correlation between neglect and sustained attention deficits. However, all patients with RHL showed generalized attentional disorders, even when no symptoms of neglect were detectable [62].

Overall, the present data highlight the role of the right hemisphere for the basic aspects of attention, such as alertness and vigilance and the involvement of both right and left hemisphere for more complex and capacity-demanding dimensions of attention selectivity. These behavioral data are in keeping with the evidence of anatomical studies [e.g., [3]]. The involvement of the right hemisphere for the basic aspects of attention was confirmed also by the acute vs. chronic patients' comparison, showing that only patients with RHL suffered from long-lasting alertness deficits. Note that in the present study, we did not examine the ability of sustained attention in patients with unilateral lesions; then, future studies will be necessary for examining this attention process.

In conclusion, the results of our study underscore the importance of a comprehensive assessment of attention functioning in stroke survivors and shield some light on different clinical and lesional variables that modulate the severity and phenomenology of attention deficits in this population.

## Figures and Tables

**Table 1:** Demographic and clinical features of patients.

	All patients (*N* = 204)	Lesion group		Control group (*N* = 42)	
		Patients with RHL (*N* = 108)	Patients with LHL (*N* = 96)		
*Demographic features*					
Age (mean ± S.D.)	62.8 ± 10.6	64.06 ± 9.1	61.53 ± 11.9	61.6 ± 9.2	*F*_(1,233)_ = 1.00, n.s.
Education (mean ± S.D.)	8.6 ± 4.5	8.12 ± 4.4	9.14 ± 4.6	9.2 ± 3.9	*F*_(1,233)_ = 1.01, n.s.
Gender (male/female)	126/78	69/39	57/39	20/22	*X* ^2^ = 5.11, n.s.
Onset (mean ± S.D.)	122.7 ± 93.1	119.2 ± 92.5	125.57 ± 92.7		*F*_(1,205)_ = .27, n.s.
*OCSP*					
TACI	35 (17.2%)	16 (14.8%)	19 (19.8%)		*X* ^2^ = .40, n.s.
PACI	117 (57.3%)	63 (58.3%)	54 (56.2%)		*X* ^2^ = .00, n.s.
LACI	45 (22.1%)	25 (23.1%)	20 (20.8%)		*X* ^2^ = .09, n.s.
POCI	7 (3.4%)	4 (3.7%)	3 (3.1%)		*X* ^2^ = .03, n.s.
*Neuropsychological correlates*					
Neglect		42 (38.9%)	/		
Aphasia		/	77 (80.2%)		

RHL/LHL: right/left hemispheric lesion; age and education in years; onset in days.

**Table 2:** Percentage of patients showing pathological performance, i.e., a normalized score at or below the fifth percentile according to normative data [39].

	All patients	Patients with RHL	Patients with LHL	*X* ^2^	*p*
Alertness without warning (RTs)	45%	57%	31%	12.94	<.001
Alertness with warning (RTs)	44%	58%	29%	16.25	<.0001
Go-No Go (RTs)	47%	49%	47%	.04	n.s.
Go-No Go (false responses)	41%	40%	43%	.14	n.s.
Divided Attention (RTs)	40%	40%	40%	.02	n.s.
Divided Attention (omissions)	71%	86%	55%	15.70	<.0001

**Table 3:** Attention performances for healthy subjects and the entire sample of patients and separately for right and left brain damaged patients (mean ± S.D.). Note that the mean RTs refer to RTs to each test, while errors refer to false responses in the Go-No Go test and omissions in the Divided Attention task.

	Patients with RHL (*N* = 111)				Patients with LHL (*N* = 97)				Healthy subjects (*N* = 42)			
	Alertness		Go-No Go	Divided Attention	Alertness		Go-No Go	Divided Attention	Alertness		Go-No Go	Divided Attention
	Without warning	With warning			Without warning	With warning			Without warning	With warning		
RTs (means)	379	336	638	787	314	287	641	724	300	291	608	724
SD	306	124	153	218	191	141	200	176	51	61	83	79
Errors (mean)	—	—	4.7	10.9	—	—	3.3	5.0	—	—	1.6	3.2
SD	—	—	5.9	7.4	—	—	3.5	4.0	—	—	2.6	2.8

**Table 4:** Percentage of patients showing pathological performance (i.e., a normalized score at or below the fifth percentile according to normative data; [39]) as a function of lesion site and presence of neglect (N^+^ = presence and N^−^ = absence) or aphasia (Aphasia^+^ = presence and Aphasia^−^ = absence). Values indicate percentages of pathological patients for each group and attention measure. Chi-square tests were computed on raw data (i.e., the number of patients with pathological performance for each sample).

	Patients with RHL			Patients with LHL		
	N^+^ (*n* = 42)	N^−^ (*n* = 69)	*X* ^2^	Aphasia^+^ (*n* = 77)	Aphasia^−^ (*n* = 20)	*X* ^2^
Alertness without warning (RTs)	74%	46%	6.93^∗^	34%	20%	0.84
Alertness with warning (RTs)	71%	49%	6.38°	34%	10%	3.29^#^
Go-No Go (RTs)	67%	38%	7.30^∗^	57%	10%	12.06^∗∗^
Go-No Go (false responses)	41%	39%	0.02	44%	33%	0.54
Divided Attention (RTs)	51%	34%	2.33	42%	33%	0.14
Divided Attention (omissions)	97%	79%	4.43°	57%	45%	0.50

^#^ *p* = .07, °*p* < .05, ^∗^*p* < .01, and ^∗∗^*p* < .001.

**Table 5:** Mean RTs and errors for each attention measure as a function of laterality and presence of neglect (N^+^ = presence and N^−^ = absence) or aphasia (Aphasia^+^ = presence and Aphasia^−^ = absence); SDs are presented in brackets.

	RHL			LHL		
	N^+^ (*n* = 42)	N^−^ (*n* = 69)	N^+^ vs. N^−^ comparison	Aphasia^+^ (*n* = 77)	Aphasia^−^ (*n* = 20)	Aphasia^+^ vs. aphasia^−^ comparison
Alertness without warning (RTs)	460 (154)	364 (114)	*F*_(1,108)_ = 15.69^∗∗∗^	349 (177)	308 (70)	*F*_(1, 93)_ = 1.82
Alertness with warning (RTs)	451 (150)	349 (104)	*F*_(1,108)_ = 17.50^∗∗∗^	334 (167)	302 (87)	*F*_(1, 93)_ = 1.16
Go-No Go (RTs)	772 (141)	674 (135)	*F*_(1,105)_ = 13.14^∗∗^	722 (171)	646 (83)	*F*_(1, 88)_ = 4.27^#^
Go-No Go (False responses)	5.5 (6.7)	4.1 (5.3)	*F*_(1, 75)_ = 1.29	3.6 (3.7)	2.4 (1.9)	*F*_(1, 59)_ = 1.89
Divided Attention (RTs)	904 (154)	852 (197)	*F*_(1, 89)_ = 2.38	829 (147)	780 (112)	*F*_(1, 81)_ = 2.37
Divided Attention (omissions)	13.5 (6.3)	9.1 (7.4)	*F*_(1, 71)_ = 6.16^∗^	5.2 (4.0)	4.1 (3.8)	*F*_(1, 57)_ = 2.08

^#^ *p* < .05, ^∗^*p* < .01, ^∗∗^*p* < .001, and ^∗∗∗^*p* < .0001. Note that covariates did not reach the significance level in any analysis.

## Data Availability

The data used to support the findings of this study are available from the corresponding author upon request.
